# Supplementary material for: An assessment of critical thinking in the Middle East: Evaluating the effectiveness of special courses interventions
Source: PLoS One. 2021 Dec 31;16(12):e0262088. doi: 10.1371/journal.pone.0262088 (PMC8719682; doi:10.1371/journal.pone.0262088)
Supplement: S1 Appendix — (DOCX) [file pone.0262088.s001.docx]

**S1 Appendix**

**CCP-SLO(5): Think critically and creatively in a variety of methods in order to make decisions and solve problems.**

| **Criterion** | **Excellent**  **(4)** | **Good**  **(3)** | **Fair**  **(2)** | **Poor**  **(1)** |
| --- | --- | --- | --- | --- |
| **Explanation of Issues**  **[Weight: 25%]** | States the issue or problem very clearly, showing full understanding. | States the issue or problem clearly, showing sound understanding. | States the issue or problem, showing limited understanding. | States no issue/problem or attempts to state the problem showing little understanding. |
| **Strategy**  **[Weight: 25%]** | Uses a solution technique, which is among the most efficient possible for the problem. | Uses a solution technique that works, but it is not among the most efficient possible for the problem. | Uses a solution technique with an acceptable connection to the details of the problem. | Uses no solution technique, or a technique with little or no connection to the details of the problem. |
| **Implementation**  **[Weight: 25%]** | Performs a sequence of logical steps leading toward the solution, without errors. | Performs a sequence of logical steps leading toward the solution, but with few errors. | Performs a sequence of logical steps, with some errors and the work is not complete. | Demonstrate no effort to implement the strategy or attempts to perform some steps but with multiple errors. |
| **Conclusion**  **[Weight: 25%]** | Conclusion is logical and correct, and clearly takes all appropriate information into account. | Conclusion is correct, but does not comprehensively take all appropriate information into account. | Conclusion is correct, but drawn without adequate support or argumentation. | Conclusion is incorrect or no conclusion is reached. |
