# Supplementary material for: An assessment of critical thinking in the Middle East: Evaluating the effectiveness of special courses interventions
Source: PLoS One. 2021 Dec 31;16(12):e0262088. doi: 10.1371/journal.pone.0262088 (PMC8719682; doi:10.1371/journal.pone.0262088)
Supplement: S2 Appendix — (DOCX) [file pone.0262088.s002.docx]

**S2 Appendix**

**Translation of the Two Questions Instrument**

1. A businessman took a 9200 Qatari Riyals loan from a bank. A four-year repayment plan with an annual interest equal to 5%. You are required to calculate the amount of interest. Please show your explanation, strategy, implementation, and conclusion (answer) as fully as possible.
2. A refrigerator is priced at 1850 Qatari Riyals. The owner of the store decided to run a 20% sale on it. What is the new price of the refrigerator? Please show your explanation, strategy, implementation, and conclusion (answer) as fully as possible.
