# Supplementary material for: An assessment of critical thinking in the Middle East: Evaluating the effectiveness of special courses interventions
Source: PLoS One. 2021 Dec 31;16(12):e0262088. doi: 10.1371/journal.pone.0262088 (PMC8719682; doi:10.1371/journal.pone.0262088)
Supplement: S3 Appendix — (DOCX) [file pone.0262088.s003.docx]

**S3 Appendix**

**The Special Course Syllabus**

|  | | \| **CORE CURRICULUM PROGRAM**  **Mathematics in Society- Math 1050**  **Spring 20172018**  3 credit hours/ 4 contact hours  Medium of instruction: Arabic & English \| \| --- \| | | |
| --- | --- | --- | --- | --- | --- |
| LECTURER | | | | |
| Name: | | Office:  Office Hours:  Telephone:  Email: | | |
| COURSE DESCRIPTION | | | | |
| \| Mathematics in Society is a math course that meets the needs of students majoring in non-science undergraduate programs not requiring calculus or discrete mathematics. The course develops students’ mathematical understanding through an engaging and non-traditional design that connects theoretical math curriculum to daily life examples and applications regarding the local context. The course emphasizes captivating topics that both effectively and creatively convey the fundamental mathematical concepts to students. Specifically, the content focuses on improving students’ numerical, statistical and logical reasoning skills through hands on activities, real life scenarios, and computer applications. \| \| --- \| | | | | |
| COURSE OBJECTIVES | | | | |
| \| The course is designed to   - Develop students’ understanding of fundamental math concepts with daily life examples and applications regarding the local context. - Provide examples of how mathematical concepts exist within daily life, work life, and universe. - Extends students’ numerical reasoning skills with real life applications and scenarios on budget planning. - Enhance students’ statistical reasoning skills with real data sets from Qatari context using computer applications and hands on activities. - Increase students’ logical reasoning skills and sense making with mathematical arguments and different types of statements. \| \| --- \| | | | | |
| STUDENT LEARNING OUTCOMES | | | | |
| \| By the end of the course, students will be able to:  1. Explore general concepts of mathematics in a manner that reveals their values in life.  2. Describe real life examples connecting fundamental mathematical concepts with daily practices.  3. Discuss how mathematical concepts are practiced within different disciplines.  4. Give examples of how mathematics exist in the universe.  5. Solve problems related to natural numbers, real numbers, integers, rational/irrational numbers, and percentages related to real life situations.  6. Describe various applications of money management with real life examples including taking control of finances, saving plans and investments, discounts and price hikes, zakat, inheritance, asset depreciation and appreciation.  7. Practice doing budget planning on real life scenarios.  8. Solve problems related to organizing data, picturing data, and finding measures of average, variation, and position.  9. Organize and picture real data sets using calculators, Excel sheets, and tablets.  10. Apply logical reasoning to interpret mathematical arguments and different types of statements. \| \| --- \| | | | | |
| TEACHING STRATEGIES | | | | |
| - Problem Solving - Cooperative Learning Strategies - Classroom Discussions - Critical Thinking - Visualization - Think Aloud - Think-Pair-Share | | | | |
| COURSE CONTENT | | | | |
| **Week** | **Course Themes** | **Dates** | **Topics** | **Content** |
| 1 | Introduction  Fun Math | 11-15, Feb | - Course Introduction  - Math Games | -Students write a reflection paper about how they perceive math and math learning (before taking the course).  -Introduce the course objectives, content, assessment, grading, policies and procedures.  -Provide course syllabus and rubrics.  -Students play math games in groups (Tangram, Geoboard, Sudoku, Sumoku) |
| 2 | Math Around Us | 18-22, Feb | Mathematics in Daily Life | Provide real life examples connecting fundamental mathematical concepts with daily practices.  (Cooking, Shopping, Travelling, Voting, Diet, Banking, Managing Time, Decoration, Population Growth) |
| 3 | Math Around Us | 25 Feb – 1 March | Mathematics in Work Life | Provide selected examples of how mathematical concepts are practiced within different disciplines  (Engineer, Doctor, Architect, Scientist, Meteorologist, Nutritionist, Artist, Athlete, Animator, Interior Designer, Car Designer, Actuary, Game Designer, Economist, , Cryptanalyst, Statistician, Photographer, Ecologist) |
| 4 | Math Around Us | 4-8, March | Mathematics in Universe | Provide selected examples connecting mathematical concepts with universal realities. (Golden Ratio, Tessellations, Dilations, Patterns, Fibonacci Numbers, Rotations, Symmetry, Fractals, Pi, Spirals) |
| 5 | Student Projects | 11-15, March | Individual Posters on Math Around Us | Students display posters prepared individually about one of the “math around us” topics ( Mathematics in daily life, Mathematics in work life, or Mathematics in universe) |
| 6 | Consumer Math | 18-22, March | Numerical Reasoning  (Theory with real life examples) | -Natural Numbers,  -Real Numbers,  - Integers  -Rational/Irrational Numbers,  - Percentages |
| 7 | Consumer Math | 25-29, March | Mathematics with Technology | - Operations priorities  - Calculations by using calculators  - Brief introduction to Excel |
| 8 | Consumer Math | 1-5, April | Managing Money  (Theory with real life examples) | - Taking control of your finances  - Saving plans and investments  - Discounts and price hikes |
| 9 | Consumer Math | 15-19, April | Managing Money  (Theory with real life examples) | - Zakat  - Inheritance  - Asset(land, housing, car, stock) depreciation and appreciation |
| 10 | Consumer Math | 22-26, April | Student Projects | Students work in groups and prepare a project about a doing a budget plan on a real life scenario. |
| 11 | Statistics in Society | 29 April – 3 May | Statistical Reasoning  (Theory with real life examples) | - Fundamentals of Statistics  - Gathering and organizing data  - Picturing Data (Tables and Graphs)  - Measures of Average, Variation, and Position |
| 12 | Statistics in Society | 6-10, May | Statistical Reasoning  (Theory with real life examples) | - Graphics in the Media  - Should you believe a statistical study?  - Misuses of Statistics |
| 13 | Statistics in Society | 13-17, May | Statistical Reasoning  (Application with IT)  Individual Reports on Statistics in Society | - Organize and Picture real data sets from Qatari context by using calculators, Excel sheets, and tablets (drawing basic tables, charts and plots)  - Students gather data on a real life situation (such as student scores, months of birth, student weights, etc) and prepare a report about how they organize the data in excel (including finding mode, median, range, and creating charts, bar graphs, or line graphs). |
| 14 | Making Sense with Math | 20-24, May | Logical Reasoning  (Theory with real life examples) | -Statements and Quantifiers  -Truth Tables  -Types of Statements |
| 15 | Ending  Fun Math | 22-31, May | -Wrap up  - Math Games | - Classroom Discussion  -Discuss the overall content  -Discuss which topics students find the most interesting.  -Discuss the course design and possible improvements  -Students write a reflection paper about how they perceive math and math learning (after taking the course)  - Students play math games in groups (Math Board Games, games of dots, chess, three sticks) |
