# Supplementary material for: An assessment of critical thinking in the Middle East: Evaluating the effectiveness of special courses interventions
Source: PLoS One. 2021 Dec 31;16(12):e0262088. doi: 10.1371/journal.pone.0262088 (PMC8719682; doi:10.1371/journal.pone.0262088)
Supplement: S4 Appendix — (DOCX) [file pone.0262088.s004.docx]

**S4 Appendix**

**Informed Consent**

You are invited to participate in a research study titled **“**An Assessment of Critical Thinking in the Middle East: Evaluating the Effectiveness of Quantitative Reasoning-Based Interventions**”**. This study is being conducted by Dr. Wael Yusuf, a core curriculum faculty member at Qatar University, to investigate the effectiveness of a quantitative reasoning course in enhancing critical thinking among students.

Participation in this study is entirely voluntary at all times. You can choose not to participate at all or to leave the study at any time. Regardless of your decision, there will be no effect on your relationship with the researcher or any other negative consequences. You will get any personal benefits or incur costs for your participation.

You are being asked to take part in this study because you are enrolled in the quantitative reasoning course. If you agree to participate, you will be asked to complete a two questions assessment during the first week of the semester, and at the end of the term. The assessment will be used to evaluate your critical thinking knowledge, skills, and abilities. This assessment should take around 10 minutes to complete. The assessment will be administered and collected by your instructor. You will submit a written answer on a piece of paper distributed by the instructor.

All of your responses to this assessment will remain anonymous and cannot be linked to you in any way. No identifying information about you will be collected at any point during the study, and your assessment will be identified only with a code developed by the researcher. Once you submit your completed assessment, there will be no way to withdraw your responses from the study because the assessment contains no identifying information.

Study data will be kept in a digital data sheet format on the researcher’s personal laptop protected by a password. Access to data will be protected on a password authorized folder, as well as file. Only the researcher will have access to the data.

There are no risks associated with this study. While you will not experience any direct benefits from participation, information collected in this study may benefit others in the future by helping to improve critical thinking education at the college level.

If you have any questions regarding the study or this research project in general, please contact the principal investigator, Dr. Wael Yousef, at wyousef@qu.edu.qa.

Dr. Wael Yousef

Core Curriculum Program Faculty

Qatar University
