# Supplementary material for: An assessment of critical thinking in the Middle East: Evaluating the effectiveness of special courses interventions
Source: PLoS One. 2021 Dec 31;16(12):e0262088. doi: 10.1371/journal.pone.0262088 (PMC8719682; doi:10.1371/journal.pone.0262088)
Supplement: Attachment — Submitted filename: Response to Reviewers.docx [file pone.0262088.s006.docx]

**Response to Reviewers**

**Comment #1**

The topic addressed in this manuscript is relevant and permeates current discussion on ways to prepare students to face labor, economic, and social changes. Although the manuscript’s focus is on ways to assess critical thinking, both the introduction and literature review sections include statements that refer to different ways to conceptualize the same theme (critical thinking) and to better situate the study it seems important to include a clear characterization of what conceptualization is endorsed in this research. In particular, the discussion of students’ development of critical thinking across different domains and its relation to the study of specific domain such as mathematics.

**Response #1**

The reviewer is asking for “a clear characterization of what conceptualization is endorsed in this research”. To satisfy this request, the following text was added in the literature review section:

*This analysis subscribes to the characterization of critical thinking as a universal set of abilities applicable across disciplines [17, 29]. The conceptualization of critical thinking as the abilities to organize information in meaningful ways, construct clear arguments based on available input, and back explanations with empirical evidence is invariant across subjects, educational levels, and contexts [30]. Critical thinkers have been shown to apply these skills in English, mathematics, science, the social sciences, and arts [6]. The current manuscript presents the argument that critical skills is generalized across problem sets, assignments, tasks, and activities common in higher education systems across countries [17].*

**Comment#2**

The section “assessment of critical thinking skills among Arab College students” is tedious and offers little information on what those assessments really mean in terms of students’ critical thinking behaviors. This section could be reduced and restructured to address and discuss what those students’ results from different tests (and versions) contributed to actually frame the study. In particular, the extent to which these test results helped to structure and implement the course.

**Response #2**

The section is completely rewritten, restructured, and reconstructed. It is shorter and clearer. It discusses the studies’ findings concerning students’ critical thinking behaviors as suggested by the reviewers. The following text was added in place of the previous section:

*Arab college students lack sufficient critical thinking behaviors. Salih [19] found low aptitude levels among a large sample of Iraqi students in the areas of information organization, arguments construction, and using empirical evidence in constructing explanations. The author suggested that students have not been exposed to critical thinking instruction, and were largely expected to memorize content. Similarly, Al-Hadabi and Al-Ashwal [20] reported low abilities in the identification of relevant information, writing coherent arguments, and justifying constructed arguments among a large sample of Yemeni students. The authors concluded that instruction in Yemeni higher education does not foster autonomous research skills.*

*Empirical evidence has indicated that Arab college students suffer from low synthesizing abilities. Al-Ziq [21] found that Jordanian students exhibit insufficient abilities in recognizing patterns, themes, and typologies when presented with complex sets of information. By the same token, Al-Bjaidi [22] found pervasive inconsistencies in female Saudi students abilities in identifying significant, relevant, and crucial information in independent research projects. Al-Mahrooqi and Denman [12] concluded that Omani students at the largest research institution in the country possess unacceptable skills in categorizing, classifying, and meaningfully coding similar information.*

*Empirical research on Arab college students critical thinking skills has found diminished abilities in information interpretation and application. Merrifield [23] examined a small sample of bilingual Lebanese students reporting low levels of logical thinking, as well as probabilistic reasoning. Similarly, Shaaban [24] surveyed a large sample of Lebanese students finding low levels of information evaluation, inspection, and detection abilities. In sum, both studies have concluded that public universities and colleges in Lebanon fails to teach students how to think critically and apply conclusions to appropriate domains.*

*Assessments of critical thinking abilities among Arab college students have noted weak research skills [21, 22]. Arab students across countries and domains have been consistently found to lack skills in questions and hypothesis formulation [19, 20]. Further, Arab college students have developed inadequate data analysis, collection, and organization abilities [20, 21]. By the same token, studies have concluded a noticeable deficiency in coherent organization of ideas, evidence, and arguments in assignments requiring extensive writing. Authors have blamed traditional instructor-centered teaching methods focusing on memorization rather than critical reasoning and problem solving [23, 24].*

**Comment #3**

It the section “instructional methods fostering critical thinking”, it is not clear how the review of this section really contributed to frame the course that was used to assess the students’ critical thinking. Indeed, it seems important to document the extent to which issues such as non-traditional instructional methods, game-based learning, independent and self-regulated learning, etc. were considered during the development of the course sessions.

**Response # 3**

This section was rewritten, restructured, and linked with how the course was developed for the study. The following new section was added to the revised manuscript:

*This section outlines the effective instructional methods found to positively influence critical thinking skills among college students. Note that this investigation built a course featuring the best evidence teaching practices reported by past research. These included collaborative, participatory, and independent learning [49, 50]. Further, the course offered students rich opportunities to practice their critical thinking skills through deliberation, collaboration, and reflection [51, 52]. The proposed course allows students to utilize critical thinking skills through interacting with peers, games, and real-world problems, as well as research projects [55, 56].*

*The proposed course feature non-traditional instructional methods found to foster critical thinking skills among college students. Lee, Lee, Gong, Bae, and Choi [40] reported standardized mean differences of 0.42 and 0.29 in favor of non-lecturing methods compared to lecturing techniques on the California Critical Thinking Dispositions Inventory (CCTDI) and the California Critical Thinking Skills Test (CCTST) in their meta-analysis. The authors concluded that the use of interactive participatory teaching allows students to take control of their own learning and offer new enriched learning opportunities across their college courses [40]. By the same token, Miterianifa, Trisnayanti, Khoiri, and Ayu [41] concluded that problem-based learning in science courses improved critical thinking abilities significantly by reporting large effect sizes of 1.2 (standardized means' difference) in their meta-analysis. They recommended college instructors to present students with puzzles, mysteries, and conundrums across subjects piquing their interest to piece a narrative together solving these problems making students constantly engaged in critical thinking activities. The current course has utilized problem-based learning activities, as well as collaborative assignments making students exercise the varying facets of critical thinking.*

*The proposed courses offers students to work with real-world scenarios, problems, and challenges proven to be linked with enhanced critical thinking abilities [3]. Abrami, Bernard, Borokhovski, Waddington, Wade, and Persson [3] concluded that exposure to real-world problems, collaborative, and deliberative learning, and mentoring all significantly increase students' appreciation and mastery of critical thinking in an extensive meta-analysis. The proposed course has assigned students to work together in-class, as well as outside to solve classical problems facing people in the real-world. This required students to work in groups, which have been linked to improved levels of problem solving and critical thinking [42].*

*The proposed course has incorporated elements of the game-based learning found to improve critical thinking skills across disciplines at the college level [43, 45]. Chen, Tseng, and Hsiao [43] performed a meta-analysis on digital games' effects on language learning, reporting moderate to substantial gains students accrue from using adventurous, challenging games in their vocabulary retention, reading comprehension, and ability to connect varying pieces of evidence to formulate a clear linguistic argument. The proposed course features the use of in-class games, as well as at-home play on digital, as well as paper-based games. By the same token, Qian and Clark [44] reviewed 29 empirical articles investigating the effects of games on 21^st^-century skills, critical thinking dispositions, and abilities, reporting moderate, consistent positive associations between varying games' features (challenge, strategies identification, implementation of techniques, and discovery) and students' mastery of critical thinking skills. The course offers students many opportunities to practice their information identification, strategic problem solving, and implementation of these solutions in an embedded environment using digital and non-digital games.*

*The proposed course in this study to foster critical thinking features the use of autonomous, as well as self-regulated learning, a method associated positively with critical thinking abilities [31]. Means, Toyama, Murphy, Bakia, and Jones [46] concluded in their meta-analysis of more than a thousand studies on online learning that courses featuring blended learning characteristics like independent and autonomous projects generate better learning outcomes and metrics. The proposed course offers students many opportunities to practice their independent research skills including formulating of research questions and hypotheses, as well as collecting evidence to test them and answer the proposed inquiries. Dunst, Trivette, and Hamby [47] reviewed 58 randomized controlled trials investigating the best adult learning method finding that those featuring searching, evaluating, using, synthesizing, and reflecting on information independently yielded the highest gains in learning output. The proposed course makes students interact with activities requiring them to evaluate, synthesize, and reflect on materials and activities. The course allows students to be in charge of their own learning through making them independent learners, which fosters critical thinking abilities [48].*

**Comment # 4**

The research design is weak, and it might be improved if a qualitative approach is added or considered to analyze the instructional approach that supported the course activities and the participants’ thought process. The questions to assess students’ critical thinking are not robust enough to actually capture what a critical thinking involves. Showing the corresponding “instrument validation” through the positive correlation of test hides the complexity involved in capture what is essential in students’ critical thinking. In addition, since the sample of the participants was not random, the statistic treatment is questionable. Here, specific examples of how the course activities were implemented and what problems the students worked during the sessions could provide more solid information regarding their problem-solving behaviors including critical thinking. Indeed, what the author shows in the Results section needs to be contextualized in terms of connecting what happened during the course sessions and ways they approaches the two questions and more problems.

**Response #4**

The comment has noted several issues in the research design and results sections. A number of paragraphs throughout the research design and results sections were added. The following is the added text corresponding to each raised point:

***Adding the qualitative component to the study:***

*This study is a mixed-methods research utilizing experimental, as well as focus groups methods. The experimental design investigates the effectiveness of a proposed course on a sample of Qatar University critical thinking abilities. The focus group collects information on students’ thoughts about the utilized instructional strategies during their course, as well as their conceptions of their thoughts processes progression.*

# *Qualitative Phase*

# *Focus Groups Design*

*Focus groups designs are appropriate when researchers desire to collect in-depth information concerning participants’ experiences. This research held a focus group with eight students who participated in the experimental study. The focus group featured questions about students’ thoughts on the effectiveness of the course in fostering their critical thinking skills. More specifically, students were asked to report their perceptions of the course instructional methods, and their own conceptualizations of their critical thinking progression throughout the course.*

# *Participants*

*Eight students participated in a two-hour long focus group held at Qatar University campus to gather information about their experiences during the course. Four males and four female students participated in the session. Two students were sophomores, three students were juniors, and three students were seniors. Four students were Qatari nationals and four students were non-Qatari citizens. Students’ ages ranged between 20 and 23. All participants were speakers of Arabic and reported sufficient fluency levels in English for academic purposes.*

# *Data Collection*

*A standard questions protocol was prepared to facilitate the data collection throughout the focus group. The author and two graduate assistants working for the core curriculum unit in the quality assurance department organized the focus group. An invitation to all participating students was sent requesting eight students’ participants for the study. Once eight students responded with agreeing to participate in the focus group, the graduate students provided them with details on the location, and timing of the vent. The focus group session took place at a conference room at Qatar University campus after the conclusion of the semester featuring the course in the experimental study. Every participant signed a written informed consent form agreeing to the terms and conditions of the research. Confidentiality was assured, and the IRB committee approved the study. The graduate assistants recorded and transcribed the responses of all eight participants.*

*Questions on the focus group focused on students’ evaluations of their experience. First, students were asked to rate their course using narratives. Second, students were asked about how the course influenced their critical thinking skills. Third, students were asked to cite specific examples and illustrations that helped them achieve better critical thinking abilities. Students were questioned about their opinions of specific instructional methods employed during the course. Finally, students were asked whether they would recommend the course to others, and their reasoning behind their answers.*

# *Data Analysis*

*Thematic analysis was used to analyze the responses of the focus group session. Inductive reading of responses informed the construction of themes. Each response was read on its own and themes were constructed. Then, all responses were read altogether to refine the constructed themes. This step resulted in merging, collapsing, and organizing themes to account for the variation in individual responses. A final set of themes was constructed. The graduate assistants analyzed the information in a similar fashion to ensure the dependability and credibility of results obtained.*

*Table 7 presents the focus groups’ thematic analysis results. Notice that four overarching themes define the responses of the eight participants in the focus group. These are: satisfaction, engagement, enjoyment, and relevance. Students highlighted their satisfaction with the course. They indicated that the materials were relevant, engaging, and enjoyable. Students noted the engagement of the instructor, the content, and their peers. Students appreciated the use of real-world applications, autonomous learning, self-directed study, and most important collaborative projects decussating collaborative and deliberative elements.*

**Table 7** Students’ Perceptions of the Course

| **Theme One: Satisfaction**  Student Two: “Overall, this course was well organized, and kept me interested. I can say that I would recommend it to my friends because I am satisfied”.  Student Five: “Looking at everything together, this course gave me a nice experience. I was satisfied with the instructor who put me first, and truly listened to what I had to say. The instructor helped me with many activities. Now, I am satisfied with my skills in information identification, organization, and presentation”.  Student Seven: “I am content with the activities we performed throughout the semester. They were challenging. They were satisfying. I had a great deal of conversation with my peers that satisfied my cravings for collaboration and deliberation”. | **Theme Three: Enjoyment**  Student Three: “I enjoyed the puzzles. I loved the mysteries. The instructor had an enjoyable style of telling stories. I learned how to make my talk more fun and engaging. This course was not only about math, it was teaching us how to enjoy doing math for life applications”.  Student Six: “The most thing I liked about the course was working with peers. I learned a lot from my classmates. They helped me when I could not understand something. I really enjoyed getting to know people, and I am friends with them now. They help me pick courses and make me a better thinker”.  Student Eight: “Taking all things considered, the class was enjoyable. I liked the mystery games. I needed to find the clues, stick them together, and present a holistic story backed by good argumentation. I want to become a forensic criminal justice professional. This course was about math, but it taught me how to think deeply”. |
| --- | --- |
| **Theme Two: Engagement**  Student One: “There was no single session throughout the semester where I felt inactive. In every class period, the instructor had us work on something. This kept everyone preoccupied and engaged with the materials, activities, and the instructor”.  Student Four: “I was doing a task for most of the time I spent in class. Even at home, I had something to do. The instructor tried his best to keep everyone busy working on something. From my observation, every student around me was engaged either in doing a problem or with a peer to solve a problem”.  Student Eight: “I felt overwhelmed at times during the semester. I was working on a project, puzzle, game, or a mystery. The course kept me working all the time. I worked hard for this course. I truly could say that I was engaged with all my senses”. | **Theme Four: Relevance**  Student One: “Everything we worked on was from real life. The instructor gave us examples from dealing with vendors to having money deposited in the bank. We tried to interact with real statistical data to formulate hypotheses and questions to be answered. I feel that the instructor made a special effort to make the course materials as real as possible”.  Student Four: “The real-world independent project taught me how to actually examine real-world issues. I practiced how to formulate hypotheses, think of relevant data sources, their reliability, and make arguments based on the”.  Student Six: “I liked the investing examples provided by the instructor. I also enjoyed trying to practice my investment skills using hypothetical scenarios based on real world stories. I wanted to understand how loans work in real-life, and this course offered many puzzles and questions dealing with banking”. |

*The thematic analysis from participants’ responses on their critical thinking process development confirms earlier theories on critical thinking construction (Elder & Paul. 1996). Students reported low levels of reflection during their first and second weeks in the course. Student two reported “I hardly took anything seriously at the outset of the semester. I did not know how to even read the questions presented’. Once challenge is introduced to students, they begin considering thinking critically. Student six noted “once the instructor started giving us puzzles, mystries, and conundrums, we started coming up with some ideas using the information available to us”. Once challenged, students develop basic critical thinking competencies. Student three suggested that “I began making categories of information, classifications of numbers and typologies of items handed or presented to me”. Once learners are exposed to efficient ways of critical thinking practice, they become avide users of them. Student eight remarked “when the instructor showed me what a good reesarch question, hypothesis, data, reliability and validity look like in real life, I began applying those ideas in my answers”. With the facilitation of the instructor, content, and peers, students become better users of critical thinking. Student five concluded “towards the end of the class, I felt comfortable creating tables, figures, and illustrations to convey complex information and make simple arguments about them using numbers and pictures”. The critical thinking progression process goes through six stages: absent reflection, early challenge, beginning practice, continued and sustained practice, advanced user, and master teacher (thinking development (Paul & Elder, 2019). Responses of participants provide evidence supporting the proposed stage theory of critical thinking development.*

Adding more text on the validation of critical thinking instrument:

*The assessment questions of Qatari students critical thinking skills were validated by a panel of five experts teaching mathematics, English, history, computer science, and biology. All experts possessed ten years of undergraduate teaching experience at various institutions in the Arab World and abroad. The experts rated the questions on their ability to capture four key dimensions of critical thinking: identification of relevant information, synthesizing information in meaningful ways, applying strategic reasoning to address the problems or issues at hand, and surmising informed conclusions based on available evidence. The rating included three categories: 1 = does not measure all four core skills, 2= measures some of the core skills, and 3 = measures all the skills. All five experts agreed that the questions used to assess critical thinking among Qatari students measured all intended domains of critical thinking giving an average of 3 to both questions.*

Adding more explanation of how course activities took place and implemented. The following text was added to the results section:

***Course Activities and Students’ Critical Thinking Skills***

*A series of reflection exercises piqued the interest and curiosity of students challenging their non-reflective thinking habits by exercising a modicum of critical reasoning throughout the semester. Every session, students were asked to reflect on the presented topic through small groups’ discussions, as well as in writing. For instance, students were asked to provide their opinions as to why mathematics is an important field to their daily lives. Subsequent course sessions asked students to provide real-life examples from their daily interactions about mathematics applications. This series of exercises developed the beginner’s critical thinking abilities through challenging preconceptions and considering new ideas about a topic neglected by the thought processes of students.*

*Students prepared a poster connecting their ideas to convey their view on their selected real-world mathematics application. Throughout this project, students needed to formulate questions, hypotheses, collect information, organize them meaningfully, and present conclusions to classmates. Further, students needed to find reliable sources, as well as statistics to back their arguments. They needed to deliberate with their peers on how to improve their posters, and present the final product to their peers at the conclusion of the term. This project cultivated students’ skills in generating relevant inquiries, solve arising problems, build strong independent research skills, and furnish scientific inquiry conceptualization, investigation, and communications abilities.*

*Students constantly interacted with technology practicing their information gathering, organization, and analysis skills, core domains of critical thinking. Students completed a set of Excel exercises throughout the semester. They were asked to collect data from their classmates, input them into Excel, organize, and analyze them in meaningful ways. Students were asked to share their results with their peers in collaborative settings, and write short reflections on their skills development throughout the process. Students’ reflections demonstrated how their skills developed from rudimentary levels to advanced in data collection, entry, screening, and analysis.*

*Throughout the semester, students were asked to work on real-world mathematics problems, and practice them using mobile games at home. The problems contained a set of information, and inputs where students may locate the relevant material to identify a strategy, implement it, and formulate a conclusion. In each of these exercises, students were asked to identify the set of important information given to find the solution, identify the strategy, explain it in detail, and implement it to formulate a coherent conclusion backed with evidence. In their reflections, students have voiced their readiness to tackle any problem using the identification, strategy formulation, implementation, and conclusion thought process. They exhibited progressive improvements in critical thinking and problem solving core skills in mathematics, as well as other domains throughout the class.*
