# Supplementary material for: An assessment of critical thinking in the Middle East: Evaluating the effectiveness of special courses interventions
Source: PLoS One. 2021 Dec 31;16(12):e0262088. doi: 10.1371/journal.pone.0262088 (PMC8719682; doi:10.1371/journal.pone.0262088)
Supplement: Attachment — Submitted filename: Response to Reviewers.docx [file pone.0262088.s007.docx]

Revisions Letter

Comment One: First, the introduction and literature review parts includes several statements linked to equal number of references; but it is not clear what critical thinking position the author actually endorses and rely on framing the study.

Response One: The author added a paragraph in the literature review section clarifying the endorsement of a particular critical thinking position. The following is the text of the paragraph added.

The author endorses the universal view of critical thinking skills, abilities, knowledge, and dispositions [17, 29]. This analysis views critical thinking as a universal set of practices applicable across disciplines [30]. Critical thinking could be taught and learned at the college level in every domain, and could be transferred to the workplace, as well as daily life contexts [6]. The current manuscript presents the argument that critical skills is generalized across problem sets, assignments, tasks, and activities common in higher education systems across countries [17].

Comment Two: it lacks information regarding how the list of elements that supposedly were part of the course was actually incorporated in the course design including the type of activities that were considered to foster the participants development of critical thinking behaviors.

Response Two: The following text and table were added to demonstrate how the elements and activities in the math course fostered the core dimensions of critical thinking among students. The text was added in the research design section.

The mathematics course featured several elements and activities that fostered the development of critical thinking skills among participants. Activities like the preparation of posters, data-driven reports, reflections, and personal budgets engaged students actively in analyzing information, organizing them in meaningful ways, and constructing defensible logical arguments. Table 1 presents several activities linked with their targeted critical thinking skill featured in the mathematics course used in this investigation.

| **Course Activity and Rationale** | **Targeted Critical Thinking Skills** |
| --- | --- |
| Activity: Preparation of individual posters about a mathematics application in real-life.  Rationale: Preparation of posters require students to gather and organize information in meaningful ways. Pictures, text, and illustrations are assembled together to convey particular arguments presented in the posters. | Organizing Information |
| Activity: Preparation of individual posters about a mathematics application in real-life.  Rationale: Students seek information from their texts, readings, the web, and other sources to make their posters. They actively engage in understanding the material to construct conclusions comprising a fully fledged analysis.  Activity: Interactive in-class discussions on connecting mathematical fundamental concepts to real-life examples like shopping, diet, voting, and population growth.  Rationale: Students actively link presented mathematical concepts to their prior experiences, and analyze how both are related. Students are asked to think how the mathematical concept relate to one or more applications they experienced in their own lives. This exercise make students analyze situations, and how they compare with each other to present an analysis to their peers and instructors in their class. | Analysis |
| Activity: Writing reflections throughout the course.  Rationale: Writing reflections force students to identify an argument. Reflections require the making of several arguments by the author to present to readers. | Construction of Arguments |
| Activity: Playing games throughout the course.  Rationale: Playing games allow students to process input and generate deductions manifested in decisions during play. Students organize, analyze, and process various information to deduce conclusions affecting their moves during play. | Deduction |
| Activity: Writing reflections throughout the course.  Rationale: Writing reflections make students aware of their own assumptions. Reflections allow students to recognize their preconceived ideas about their own thoughts, beliefs, and values. They permit them to learn how their assumptions relate to others’ feelings and beliefs. | Recognition of Assumptions |
| Activity: The use of Microsoft Excel throughout the course.  Rationale: Students generate means, medians, standard deviations, variances, ranges, frequencies, and other statistical numbers to describe data. Students utilized Excel for hypothesis testing to make decisions on proposed claims. They used evidence to infer conclusions about the data at hand. | Inference |
| Activity: Data collection and analysis on a real-life topic.  Rationale: Students collect data from their peers on a particular subject. They input the data into Excel. They analyze the data using statistical models. Students are asked to interpret their results, and offer their own understanding of the patterns recognized.  Activity: Preparing budget plans.  Rationale: Students input information into Excel about their spending to better understand their consumption habits. They make conclusions, and interpret them in light of their own lives. They also offer feedback to other peers on their budget plans, and how they could improve their spending patterns with clear interpretations of the evidence presented. | Interpretation |
| Activity: Collaborative small group discussions and assignments.  Rationale: Students are presented with several arguments authored by their peers. They need to evaluate the accuracy, precision, reliability, and validity of the arguments. They begin to judge the merit of arguments based on criteria learned throughout the course or from their prior knowledge. They need to defend their arguments on assessing others’ thoughts and beliefs. | Evaluation of Arguments |
| Activity: Data collection and analysis.  Rationale: Students are asked to explain the trends in their own collected data. They are asked to clarify the patterns observed, and offer plausible logical arguments explaining the observed themes in the data.  Activity: Preparation of budget plans.  Rationale: Students are asked to explain why they spend on the goods and services presented in their budgets. They are also asked to explain how their future plans improve their consumption habits to enhance their budgeting skills. | Explanation |
| Activity: Preparing of individual posters on mathematics applications in real-life.  Rationale: Students gather graphical, tabular, and illustrative empirical evidence about their topic. They use the various information in the empirical documents to defend their arguments.  Activity: Students practice on Microsoft Excel.  Rationale: Students enter new information in Excel sheets to present empirical information on given topics. Students utilize percentages, frequencies, descriptive statistics, and graphs to convey basic conclusions about their topics using empirical data. | Use of Empirical Evidence |

Comment Three: there is no information regarding the pre-test questions and its possible connection with the activities that were implemented during the course.

Response Three: The following text was added to the research design section.

Note that the pre-test and post-test questions were the same presented in Appendix B. Students’ scores on the pre-test are unrelated to course activities. Students have not completed any course activity prior to being pre-tested on critical thinking. They were unaware of activities to be completed throughout their course. The pre-test scores were reflections of students’ baseline critical thinking abilities.

Comment Four: Indeed, the two questions seem limited to actually assess the participants' critical thinking competencies.

Response Four: In the research design section, a correlation of 0.80 was reported between scores on the two questions’ instrument and a widely used validated critical thinking appraisal. A correlation of 0.80 is considered a strong indication of internal and external validity of the two questions instrument.

Comment Five: The qualitative part results offer little information about the participants' actual answers and their relation to their critical approach to deal with them. The qualitative part focused mainly on the participants perceptions about the course without exploring their ways of thinking to deal with those questions.

Response Five: The following text was added to the results section.

The course has facilitated the development of critical thinking skills and practice among participants in several respects. Students ability to question arguments and talking points has improved over the course of the semester. Participants cited starting the course with minimal questioning practice, and ending the course with high levels of inquisitiveness and curiosity. Participants’ problem-solving skills evolved from nascent to satisfactory as a function of completing the course. Many students expressed intimidation, fear, and hesitancy when faced with mathematical or statistical problems. Focus group responses revealed significant improvements in students’ abilities in identifying relevant information, unitizing problems, determining courses of actions, and following set processes to achieve reasonable solutions. Students focused on how the course improved their skills in identifying sound empirical evidence. Participants learned that randomized trails, meta analyses, systematic reviews are superior to cross-sectional and observational studies. They also learned the differences between qualitative and quantitative research, which helped them in judging the quality of evidence when they performed independent research throughout the course. Students expressed their self-reflection skills development throughout the course. They began writing their reflections without offering much insight on what they truly believe about anything they write about. By the end of the course, they developed their own voice as a result of learning how to question others’ assumptions, as well as ideas. They now have their own active voice when critically appraising others’ arguments.

Table z Improvements in Critical Thinking among Students

| Theme One: Questioning  Student One: “At the start of the course, I did not really ask many questions when I was prompted with a problem to solve. Over the course of the semester, I learned to ask meaningful questions like why did the author say whatever he or she said, and who was the audience? I started to think about the context. Did the author want to advance a specific view or not. This was helpful when I did my independent research poster project”.  Student Four: “In my collaborative discussions with peers, I used to listen more than anything else. During this course, I learned to ask critical questions. These are like how did you know this is the case? What type of evidence proponents of this argument possess? Could there be any defensible rival explanation to what they proposed? This practice helped me become aware of merits and demerits of arguments”.  Student Eight: “This course taught me how to write effectively. I now begin my essays with a set of questions. These include who said this? Why did they say it? How did they present it? Before this course, I really did not pay much attention to how I write, or even where to start. Now, I have at least one way to guide me”. | Theme Three: Evidence Evaluation  Student Five: “Before enrolling in this course, I used to accept any numbers or factual statements without questioning the source. Now, I try to look for how the data was collected, and whether there were any biases imposed on the process. I question the assumptions authors make when arguing their points of view. This helped me craft the narrative for my poster project”.  Student Six: “I now understand the difference between quantitative and qualitative evidence. I also understand the difference between observational and experimental studies. Before this course, I had no idea about what good evidence looks like. Now, I am more confident”.  Student Eight: “Prior to completing the research poster project, and collaborative discussions, I had little confidence in assessing arguments. Applying the pyramid of good evidence helps me identify what arguments are better than others. I now look for randomized trails, meta analyses, and systematic reviews for good evidence on any topic I want to learn about”. |
| --- | --- |
| Theme Two: Problem-Solving  Student Two: “When I began this course, I used to be intimidated by math or statistical problems. I used to think that I needed a great deal of skill to do them. This course taught me how to unitize problems into small pieces. I begin solving each piece on its own, and once all pieces are resolved, I weave them together to solve the bigger problem I am asked about. I achieved great success when I applied this strategy”.  Student Three: “When I signed up for this course, I did not expect to learn much. I learned one thing, which is not to panic when I am faced with a problem. I start by understanding the input or information given to me. I ask for more, if I could better understand the problem. Then, I come up with one or more solutions if applicable. I compare solutions, and find the best one. I have a process behind my problem-solving approach”.  Student Seven: “I have taken several courses at the college level mentioning problem solving as a learning goal. I have never understood the actual meaning or practice of the term. This course taught me just that. Now I know how to approach a problem. I dissect the issues into difference simple bits of information. I understand them through analysis. Then, I look how others in the past have approached the problem. I apply the best approach recommended by most”. | **Theme Four: Having an Active Voice**  Student Two: “I used to narrate what others have to say about any topic I discuss in my classes. Now, I add my opinion based on logic and evidence. I interpret what others say in view of existing evidence”.  Student Five: “In my writings and presentations, I rarely spoke out on my behalf. Now, I have more confidence in stating my view on things. I narrate what people have to say about something followed by my assessments and conjectures”.  Student Six: “I never questioned any assumptions. Now, I do. This course made me distinguish between my understanding of what others have to offer on something, and what my perspective is on the same thing. I now have the ability to connect my ideas to prior views, and justify them”. |

When asked about critical thinking strategies learned throughout the course, participants explicitly referenced information organizing methods, argument construction techniques, and evidence evaluation approaches. Students one, three, and seven all mentioned that classification, categorization, and construction of typologies to organize various information in meaningful ways are new skills they intend to use in other courses. Students two, four, and eight indicated that the use of basic statistics, empirical facts, and numbers in constructing arguments is an essential ability for college success, and one to be practiced by them even after graduation. Students five, six, and eight mentioned their improved abilities to gauge the merit of evidence using quantitative and qualitative research criteria. They emphasized the importance of methodological appraisal prior to taking any finding in a noticeable manner. Students expressed the progression of their skills in thinking critically about every task they face using the above mentioned strategies, as well as others.

**Reviewer One Comments**

Comment Six:  I found the addition of Qualitative section does not necessarily strengthen the paper, while it may be good to include them as supporting information.

Response Six: Please note that reviewer two in the previous revisions round requested the addition of a qualitative section. The qualitative section was improved as highlighted by response five above. Additional details on the focus group responses were inserted in the results section. The additions highlight the ways the course facilitated the development of critical thinking strategies among students. The addition also ensured to inform readers about the strategies students learned from the course, and would implement them in future learning opportunities.

Comment Seven: Design weakness cannot be handled with re-writing.

Response Seven: There was no explicit mentioning of any weaknesses in the above comment. All weaknesses highlighted in the previous revisions were addressed in the revised version. I am happy to clarify any issues concerning the design. Note that administrative protocols at Qatar University limit the ability of researchers to perform all desirable research activities. This is a common limitation experienced by higher education researchers. Regardless of the comment, I am happy to offer any explanation, clarification, or illustration of remaining reservations on the design of the study.

**Reviewer Two Comments**

Comment Eight: A significant number of issues have arisen with regards to the assumption that mathematics and Critical Thinking (CT) are aligned, and this discussion is missing in light of how a mathematics course has been targeted specifically for this investigation. [C.f. Inglis M, Attridge N. Does mathematical study develop logical thinking? Testing the theory of formal discipline. London: World Scientific Publishing Europe Ltd; 2016.] There needs to be a true clarity between the mathematical course content and assessment, and then the teaching style and focus on the emerging CT skills. Any link between these two themes needs to be carefully managed. The last sentence of the first paragraph of the conclusion is particularly confusing. Is the suggestion that it was the quantitative reasoning-based nature of the course that contributed to the fostering of the CT skills or the method of instruction that included game-based learning, collaborative and independent learning, real-world projects, etc.?

Response Eight: The following text was added in the research design section to clarify the connection between mathematics and critical thinking.

The mathematics course intervention was designed in light of the empirical support of the Theory of Formal Discipline (Inglis & Attridge, 2017). This theory predicts that exposure to mathematics enhances students’ reasoning, inferential, and logical skills. It is expected that if students complete several cognitively demanding activities requiring higher order reasoning abilities, they will foster better information organizing, explaining, interpreting, argumentation, and communication skills, facets of critical thinking. Table e demonstrates how the course content, teaching style, assessment, and critical thinking skills align within the logic of The Theory of Formal Discipline.

The course mandated students to prepare independent research using posters. Participants prepared personal budgets using Microsoft Excel. Students completed several game-based learning activities requiring them to identify information, explanations, and arguments. Numerous reflections were inserted helping students identify their assumptions, as well as those of their peers, and critique them. Data collection and analysis projects involving basic statistics were inserted to involve students in doing science activities. All such assessments are expected to improve critical thinking skills within a mathematics instructional setting.

Top of Form

Inglis, M., & Attridge, N. (2017). *Does mathematical study develop logical thinking?: Testing the theory of formal discipline*.

Table e Selected Course Content, Teaching Style, Assessment, and Critical Thinking Skills Alignment in the Mathematics in Daily Life Course

| Course Content | Teaching Style | Assessment | Critical Thinking Skills Targeted |
| --- | --- | --- | --- |
| Basic Mathematical Concepts: This series of lectures included various fundamental topics in mathematics. Those included: Natural Numbers, Real Numbers, Integers  Rational/Irrational Numbers, Percentages  Operations priorities, ratios, proportions, and fractions. | The real case study approach was utilized to illustrate each concept. Students were given two real-life cases involving the implementation of each topic, or more. Students were asked to review the cases individually, then in groups. Students were given the chance to clear any misconceptions with the instructor. The instructor handed the complete solutions for the two cases once discussions were over. | Each student completed two similar cases in class. Then, students convened in groups to review their answers. Once all students completed their discussions, each student submitted solutions along a short reflection of what changes were made and why to the initial solution before group disccusions convened. | Identification of relevant information, process/strategy formulation, implementation of specified process, analyzing information, explaining, and interpreting the generated outcomes. |
| Basic Statistics: Frequencies, Percentages, Central Tendency Measures, Variance, and Spread Graphs. | The use of interactive technologies like Microsoft Excel built-in Data Analysis package. The demonstration of how to use the functions in-class, as well as online. Students were given a YouTube channel with well-designed videos walking them through each function in the Data Analysis plug-in. | An individual research project was developed to assess students’ abilities in collecting, analyzing, and interpreting data using Microsoft Excel. | Organizing information, Analysis, Questioning, Hypotheses generation, Interpretation, and Explanation. |
| Mathematical Literacy: This series of lectures covered many essential concepts in mathematics for everyday life spanning across disciplines. The content included the following topics: Golden Ratio, Tessellations, Dilations, Patterns, Fibonacci Numbers, Rotations, Symmetry, Fractals, Pi, Spirals. | Collaborative discussions with each topic were convened in each lecture. Questioning was used to have every learner generate three basic questions with model answers on each topic. The instructor facilitated collaborative discussions to address any arising misconceptions. Supplementary YouTube videos were provided to students to review the material at their convenience. | Students groups’ responses were assessed by the instructor in class and feedback was immediately given. Students’ individual questions were handed to the instructor who addressed any misconceptions in writing within 2 days from submission date. | Questioning, Communication, Argumentation, Explanation, and Interpretation. |
| Basic Accounting and Finance Principles: Budgeting, Liabilities/Assets, Loans, Investments, and Taxes. | Technology-based learning facilitated by online interactive simulation tools was used throughout this portion of the course. Students used Microsoft Excel to enter, organize, and analyze their financial information. Online loans, investments, and budgeting tools were used to demonstrate how numbers change based on simple altercations of conditions. | Students were asked to prepare a personal budget using Excel. Students were asked to list their liabilities, assets, monthly consumption, and projected savings. Students reviewed their budgets in groups, and submitted their final product to the instructors. Students were asked to write a short reflection on the exercise. | Identification of relevant information, Information organizing, Categorization/Classification, Analysis/Calculation, Explanation, and Interpretation. |

Bottom of Form

The last sentence of the first paragraph in the conclusion was rewritten to become the following:

This investigation lends support to the Theory of Formal Discipline arguing that mathematics for daily life instruction improves students’ universal critical thinking skills.

Comment Nine: The Two Questions Instrument in Appendix B are financial mathematics questions. How were these unpacked as CT questions? Having some examples would be worthwhile here. Table 7 provides subjective student perceptions. A Table considering mathematical answers that present more than mathematics but the CT skills therein would be equally appropriate. How were the answers explicitly analysed vis-a-vis CT? How did the five measures get mapped out explicitly in these financial mathematics questions? This would be highly valuable in the context of the murky understanding of CT definitions, how and whether it can be taught or fostered and the issues that have presented themselves in understanding these with comparison to mathematical thinking.

Response Nine: The following text was added to the research design section.

The two questions used to assess students critical thinking skills in this investigation taps into participants’ abilities to identify relevant information, strategies for solutions, implementation of processes, and explaining their answers. Within this set of critical thinking skills, both questions demand students to analyze the information conceptually, as well as computationally. Students were expected to generate a mathematical answer along a written response demonstrating their answers. Table j shows an example of one students’ response clarifying how critical thinking skills were related to the questions. Note that the student agreed to have his response be presented anonymously.

**Question One**

The question asks about the amount of interest a businessman will pay on a loan. The amount of the loan was 9200 Qatari Riyals. The interest applied was 0.05 or five percent. The repayment period was four years. To find out the amount of interest, an application of the simple interest formula is applicable. This easy to apply calculation entails multiplying the amount of the loan with the amount of interest and the period of time. In words this translates into:

Amount of Interest = Amount of loan * Percent of Interest * period.

Amount of Interest = 9200 * 0.05 * 4 = 1840.

The amount of interest the businessman will pay is 1840 Qatari Riyals. This is somewhat high of an interest given that the amount of interest is equal to 20% of the original loan taken. I would not suggest the businessman to take out this loan. I would advise him to seek other lenders or options. My strategy was to identify the relevant interest amount formula. This was easy given the simplicity of the task. The implementation of the strategy was given by plugging the numbers into the formula. My conclusion was represented by my general recommendation to the businessman by showing him that the 1840 Riyals on a 9200 Riyals loan in Qatar is a bit high.

**Question Two**

The question asks about finding the final price of an item after applying a sale. The original price for the item was 1850 Qatari Riyals. The amount of sale applied to the item was 20%. To find the new price, two steps must be taken. First, the amount of sale removed after applying the 20% must be calculated. Second, this amount must be deducted from the original price of the item.

First Step:

1850 * 0.20 = 370

Step Two:

1850-370 = 1480

The new price for the refrigerator is 1480 Qatari Riyals. My strategy was to identify what steps are needed to solve this problem. Once I figured out the two steps I explained above, I implemented them by calculating the numbers. My advice to any customer is not to buy the refrigerator. In Qatar, 370 Qatari Riyals equal to $100. This is not a great deal on an item. Items here in the island are expensive. My advice to the seller is not apply the sale. I believe that people are willing to pay given the high salaries they receive. In Qatar, an average income for a Qatari is about $90,000. People could buy things on payments too. I would not run a sale if it was my shop.

Comment Ten:
Appendix A is important; it is also linked to Comment #2. What is “the most efficient solution strategy”? One that is quick or one that reveals an underlying framework that may facilitate further solutions in other domains? There is an underlying presumption of qualities in Appendix A that seems aligned precisely with the difficulties underlying our current understanding of CT. Showing a solution considered most efficient, versus non-efficient, versus acceptable, versus non connected, as the table partitions, would be valuable to see.

Response Ten: The following text was added to the research design section.

Table u presents two contrasting examples of acceptable and unacceptable answers to one of the critical thinking questions on the assessment. Note that students produced variations close to either of the two. Students who produced answers as detailed as the left anel of the table received an excellent rating since they clearly demonstrated how to explain the information given, identified a strategy, implemented it, and concluded a statement about the scenario. While the right panel provided the correct answer, the response did not reflect much thought and consideration to explicitly spelling out the process of finding the solution nor interpreting the generated output. Efficient strategies refer to fully detailing the courses of actions to be taken by the student or participant to reach the answer. Quick ways to find the solution are considered efficient only if the response detailed a strategy, its implementation, and an interpretation about the conclusion reached.

| Acceptable/Efficient Responses | Unacceptable/Non-efficient responses |
| --- | --- |
| Explanation: The question wants us to find out how much a businessman will pay in interest on a loan in Qatari Riyals. The loan amount was 9200. The repayment period was 4 years, and the interest rate was 5%.  Strategy: This question provides clear information about loan amount and interest rate with repayment period. There is no complex interest calculations required. This is the simple interest rate formula. I will apply the simple interest rate formula to find out how much the business man will pay in interest.  Implementation: I will plug in the numbers in the formula: Amount of Interest = Loan Amount times the interest rate times the repayment period. Given the information we have, this gives us: Amount of Interest = 9200 * 0.05 * 4 = 1840.  Conclusion: The amount of interest to be paid by the businessman is 1840 Qatari Riyals. Distributed on four years, 460 Qatari Riyals will be paid every year for four years by the businessman in interest. This amount is manageable given the low annual interest amount, about $150. I would recommend the businessman to take out the loan. | The question wants me to find the amount of interest. Here is how I find it. 9200 * 0.05 * 4 = 1840. The answer is 1840 Qatari Riyals. I applied the formula learned in class. I concluded that the interest amount is 1840. My strategy was to apply the formula. I plugged numbers in the formula and got the answer. |

Comment Eleven: Comment #4: What was the short-form Glaser-Watson test used? Can this be explicitly given and its choice corroborated? Was it the exact same questions (both Glaser-Watson and the 2 financial mathematics) used at the beginning and end of the course? If so, how can we explain an improvement as an improvement in CT and not simply learning the course content or even re-exposure? Could this be unpacked.

Does Figure 1 include data gathered at the beginning and end of course? How does it demonstrate the validity of CT assessment and not simply an education in financial mathematics? Further details could be explored here.

Response Eleven: The following text was added to the results section.

The Watson-Glaser Critical Thinking Appraisal-Short Form has forty possible points that could be converted into percentages or percentiles. The test assesses students abilities on inference, recognition of assumptions, deduction, interpretation, and evaluation of arguments. This set of abilities cover core dimensions of critical thinking including defining problems, identification of important information for finding solutions, recognition of explicit and implicit assumptions, constructing arguments/hypotheses, and drawing logical and empirical conclusions. The test is patented and cannot be reproduced without obtaining a license from the copyrighted institution. In this research, the forty items 1994 English version was translated into Arabic by a panel of higher education faculty at Qatar University. The appraisal is a set of scenarios from common life contexts like schools, workplaces, or organizations. Each scenario is followed by a number of items to be answered by participants. All items have choices ranging from 2 to 5 for test takers to select from. The total score is the sum of all items on the test. Watson and Glaser calculated the reliability coefficient for this version in 1994 and reported a Cronbach Alpha of 0.81. Note that students only took the WGCT-SF 1994 Arabic translated version prior to completion of the course to estimate the validity of the two questions’ instrument in Appendix B. Both tests were taken at the same session.

The main proposition of this analysis is that a course in mathematics using instructional strategies fostering critical thinking skills at the college level enhances students’ inference, recognition of assumptions, interpretation, strategy formulation, hypotheses construction, and explanation skills. This course is expected to improve students’ core critical thinking skills. Therefore, it is hypothesized that exposure to the materials, activities, instructional methods, and assessments in the course lead to gains in critical thinking abilities measured by Appendix B questions: explanation, strategy, implementation, and conclusion. Exposure to baseline measurements of critical thinking is thought to have minimal effects on students’ critical thinking skills. Students will not be inclined to study the tests they took prior to their course. They will likely complete the expected assignments from them during the to be taken course.

Comment Twelve: How did the present authors converge on their particular method to foster CT skills? Here lies the strength of the work. It is not clear at all how the study mentioned in [41] recommends puzzles, mysteries, and conundrums; and study [40] pertains to nursing students. Maybe this section could be shortened? Maybe the authors could celebrate their creativity here more? Giving details to frame the mathematics course as to how it was to build CT skills is of interest (while bearing in mind that how to build CT skills is still an open question).

Response Twelve: The following text was added to the discussion section.

The present findings corroborate that the combined use of collaborative, game-based, reflective, technology-based, simulation based learning strategies in mathematics for daily life courses foster universal critical thinking skills. The use of interactive collaborative discussions help students communicate their arguments in a better fashion while appraising their peers’ arguments more critically. Game-based and simulation-technology mediated learning help students become better at identifying useful information, organizing data, and better analysts of information. They also allow students to construct their own readings and understandings of the information generating creative tables, graphs, conceptual maps, and new ideas. Reflections permit students to question their own assumptions, as well as those of others. They enable students to present their ideas backed with their attempts of making cogent arguments. Amalgamating all such teaching methods while teaching mathematical and statistical literacy facilitate the development of core dimensions of critical thinking skills.

Comment Thirteen: Less seriously, the manuscript places a lot of emphasis on the Arabic world, without then truly unpacking why the Arabic world is so different to other communities. The study in question certainly does not present a global analysis. Weak mathematical students are prevalent worldwide, presenting similar issues. Are CT behaviours celebrated anywhere? The opening sentence [line 117] is a strong one. If there is a lack, this suggests a comparison. And what is “sufficient”? Line 131: What is an “unacceptable skill”?

Response Thirteen: The following text was added to the discussion section.

Arab college students possess few distinct characteristics from many of their peers around the world. In their high school education, they rarely engage in cognitively demanding activities requiring higher order reasoning skills. Arab college students have lower college readiness training and preparation compared to their peers in North America, East Asia, Europe, and Oceania (Rajab, 2018). The instruction of Arab college students rely more on the lecture-method compared to other regions around the world. Arab students are used to memorization, scripting, and limited inquiry-based tasks (Al-Zou’bi, 2021). Higher education institutions in the Arab World desire to bridge this gap by emphasizing the importance of critical thinking in their documents and practices (Al-Mahrooqi & Denman, 2020). Nevertheless, their attempts of systematically investigating salubrious interventions like the proposed course in this study are feeble. Therefore, the importance of focusing on Arab college students’ critical thinking abilities stem from the normative desire to enhance students’ performance in their academic programs, as well as their daily life practices.

Rajab, K. D. (2018). The effectiveness and potential of E-learning in war zones: An empirical comparison of face-to-face and online education in Saudi Arabia. *IEEE Access*, *6*, 6783-6794.

Al-Zou’bi, R. (2021). The impact of media and information literacy on acquiring the critical thinking skill by the educational faculty’s students. *Thinking Skills and Creativity*, *39*, 100782.

Al-Mahrooqi, R., & Denman, C. J. (2020). Assessing Students' Critical Thinking Skills in the Humanities and Sciences Colleges of a Middle Eastern University. *International Journal of Instruction*, *13*(1), 783-796.

Comment Fourteen: Typographical errors in the manuscript.

Response Fourteen: The paper was submitted to an English editing service located in Ypsilanti, Michigan, USA. The editors polished the manuscript.

Comment Fifteen: How is the third area of disagreement different from the first?

Response Fifteen: The highlighted text by the reviewer was removed in line 103. For readers, it would be best to only focus on the main points of disagreements among critical thinking experts.

Comment Sixteen: Line 155: The wording “proposed” course is confusing because it is no longer proposed but has occurred. This was confusing throughout the document.

Response Sixteen: The word proposed was removed.

Comment Seventeen: Typographical errors.

Response Seventeen: The manuscript was sent to Lighthouse Academic Services, LLC in Ypsilanti MI for editing services. All typographical errors were addressed.
