# Supplementary material for: An assessment of critical thinking in the Middle East: Evaluating the effectiveness of special courses interventions
Source: PLoS One. 2021 Dec 31;16(12):e0262088. doi: 10.1371/journal.pone.0262088 (PMC8719682; doi:10.1371/journal.pone.0262088)
Supplement: Attachment — Submitted filename: Response Letter.docx [file pone.0262088.s008.docx]

Response to Reviewers

Thank you for the opportunity to revise my paper entitled “An Assessment of Critical Thinking in the Middle East: Evaluating the Effectiveness of Special Courses Interventions” The reviewers’ comments were both helpful and insightful. I have made a serious effort to address all of the issues raised by them and think that the paper is stronger as a result. I are going to detail here what their issues were, and how I attempted to address them.

1. Please present a clean version without highlighting the changes and reduce the length of the paper to 60 000 characters including space.

I have reduced the manuscript from 100,000+ characters to less than 60000 characters (without appendices).

2. In the introduction and literature review parts, include only relevant papers that were important to support and contributed to frame the study. Avoid presenting a lists of several paper to support a single statement.

Unnecessary references were removed from the text.

3. Review and clarify how Arab College students get assessed differently in comparison with international students, one of the reviewers asked for this clarification

This was discussed on page 4-5, by adding the following text:

“There are several systematic differences between the assessment of Arab students on critical thinking compared to many students’ populations like those of North America, Europe, and East Asia. First, Arab students are exposed to less frequent, as well as systematic critical thinking appraisals. Second, Arab colleges and universities seldom evaluate their students’ abilities in critical thinking. Third, courses at both the undergraduate and graduate levels in Arab colleges and universities do not feature high levels of cognitively demanding tasks like intensive writing, experiential projects, in-class simulations, or independent research. Fourth, Arab college students have performed systematically lower on critical thinking assessments compared to their counterparts around the world. Fifth, Arab students are assessed using translated non-authentically developed instruments within their own environments leading to biased understandings of several items on critical thinking appraisals.”

4. Instead of adding texts or contents to previous sections of the old version, please review the content of sections that were asked to clarify and integrate the ideas to provide a coherent argument.

The added material in the last review were incorporated to existing text, and unnecessary text was removed.

5. Conceptualize and support the choice of only two questions to assess the participants' critical thinking and related the students' work to their course behavior while working on instructional tasks.

This was discussed on page 9, by adding the following text:

“The two questions’ instrument intended to measure four fundamental dimensions of critical thinking ability: identification of relevant information, the formulation of a strategy, the implementation of the chosen strategy, and inferring an interpretable statement based on the evidence available. Each of the question requires learners to engage in all four conceptual cornerstones of critical thinking. The use of two questions allows the assessment of critical thinking in a rapid manner without overburdening students with taking hours-long assessments. Correlations between students’ scores on the two questions and a popular appraisal yielded high associations signaling the validity of the two questions’ instrument.”

6. Avoid long tables, select relevant information and results that were used to answer research questions

Suggestions of summarizing the long tables in few sentences were incorporated. The long tables were removed from the manuscript and the appendix to ease the readability of the manuscript on interested readers

7. Focus on discussing the meaning associated with the process of instrument validation, rather than presenting results from pretest, etc.

This was addressed on page 14, by adding the following text:

“Instrument validation is needed to verify if the suggested measures capture the intended meaning. In this case, it is required to confirm if the two questions assessment is sufficient to measure participants’ level of critical thinking.”

8. Discuss the extent to which both qualitative and quantitative analysis really contributed to answer research questions and the type of limitations that were faced during the development of the study

This was addressed on pages 29 and 31, by adding the following texts:

“Mixed-methods research design provided an ideal tool to pursue this research for three particular reasons. First, it allowed for a comparison of quantitative results and quantitative findings. Second, the qualitative component, in particular, enabled the researcher to reflect participants’ point of view. Last but not the least, it encourages more scholarly engagement on the topic by inspiring methodological flexibility.” On page 29

“The difficulty in recruiting a large representative random sample limits the study’s external validity. While the sample included in the study is heterogenous representing many different segments of the population, its selection was not random nor large. The use of 80 students in a quasi-experimental design appears sufficient, however, the great variety of students in the Arab World requires a larger sample from each group in the population. In addition, Qatar University students come from highly qualified pool of college students in the Arab World.” On page 31
