# Supplementary material for: An assessment of critical thinking in the Middle East: Evaluating the effectiveness of special courses interventions
Source: PLoS One. 2021 Dec 31;16(12):e0262088. doi: 10.1371/journal.pone.0262088 (PMC8719682; doi:10.1371/journal.pone.0262088)
Supplement: Attachment — Submitted filename: Response Letter.docx [file pone.0262088.s009.docx]

Response to Editor

Thank you for taking time and paying attention to the manuscript submitted for publication to your journal. I have moderated the language reflecting the limitations of:

1. Assessment Instrument

2. Sampling Strategy

3. Difficulty in Replication

4. The extent of participants' collaboration.

Please note that this manuscript has gone through three full reviews, and many tables inserted within the manuscript were demanded by reviewers. For instance, the Qualitative section tables were requested by a reviewer in the first round.

I am sincerely thankful for your time and attention given to the manuscript. Your comments have made the manuscript shorter, concise, and organized. All these qualities are great for readers!

Thank you
